# RNA and DNA Bacteriophages as Molecular Diagnosis Controls in Clinical Virology: A Comprehensive Study of More than 45,000 Routine PCR Tests

**DOI:** 10.1371/journal.pone.0016142

**Published:** 2011-02-09

**Authors:** Laetitia Ninove, Antoine Nougairede, Celine Gazin, Laurence Thirion, Ilenia Delogu, Christine Zandotti, Remi N. Charrel, Xavier De Lamballerie

**Affiliations:** 1 UMR190, Université de la Méditerranée and Institut de Recherche pour le Développement, Marseille, France; 2 Pôle de Microbiologie and Maladies Infectieuses, Assistance Publique – Hôpitaux de Marseille, Marseille, France; 3 EHESP School of Public Health, Marseille, France; Nanyang Technological University, Singapore

## Abstract

Real-time PCR techniques are now commonly used for the detection of viral genomes in various human specimens and require for validation both external and internal controls (ECs and ICs). In particular, ICs added to clinical samples are necessary to monitor the extraction, reverse transcription, and amplification steps in order to detect false-negative results resulting from PCR-inhibition or errors in the technical procedure. Here, we performed a large scale evaluation of the use of bacteriophages as ICs in routine molecular diagnosis. This allowed to propose simple standardized procedures (i) to design specific ECs for both DNA and RNA viruses and (ii) to use T4 (DNA) or MS2 (RNA) phages as ICs in routine diagnosis. Various technical formats for using phages as ICs were optimised and validated. Subsequently, T4 and MS2 ICs were evaluated in routine real-time PCR or RT-PCR virological diagnostic tests, using a series of 8,950 clinical samples (representing 36 distinct specimen types) sent to our laboratory for the detection of a variety of DNA and RNA viruses. The frequency of inefficient detection of ICs was analyzed according to the nature of the sample. Inhibitors of enzymatic reactions were detected at high frequency in specific sample types such as heparinized blood and bone marrow (>70%), broncho-alveolar liquid (41%) and stools (36%). The use of T4 and MS2 phages as ICs proved to be cost-effective, flexible and adaptable to various technical procedures of real-time PCR detection in virology. It represents a valuable strategy for enhancing the quality of routine molecular diagnosis in laboratories that use in-house designed diagnostic systems, which can conveniently be associated to the use of specific synthetic ECs. The high rate of inhibitors observed in a variety of specimen types should stimulate the elaboration of improved technical protocols for the extraction and amplification of nucleic acids.

## Introduction

Real-time (rt) PCR and reverse transcription (RT) PCR techniques are rapid and versatile diagnostic procedures broadly used in clinical virology where there are mostly considered as diagnostic “gold standards” [1]. Monitoring rt-PCR and rt-RT-PCR assays and validation of the results rely on the use of relevant external or internal controls (ECs or ICs) [1], [2] and commercial kits including such control systems are being increasingly improved for the molecular diagnosis of a number of pathogens such as HIV, hepatitis viruses, influenza viruses etc.. However, one of the main strengths of rt-PCR is versatility, which provides the opportunity to set-up “in-house” protocols for specific pathogens. The scientific literature now includes an impressive number of ‘home made” assays for various viral agents. Whilst most commercial kits include both ICs and ECs allowing accurate validation of the results [3], “home made tests” are frequently performed in the absence of ICs and therefore without any possible individual monitoring of each diagnostic reaction. For example, the detection of technical errors or PCR amplification inhibitors is intrinsically impossible if only ECs are used. In addition, ECs are usually undistinguishable from the native genome.

Here, our objective was to develop and test on a large number of clinical samples a bacteriophage-based IC system suitable for a standard laboratory of medical virology. We present results obtained by using T4 and MS2 bacteriophages as ICs in a routine-based evaluation including 8,950 clinical specimens, representing 36 types of samples, submitted for PCR detection of selected viruses including DNA viruses (*Herpesviridae*, JC and BK viruses, parvovirus B19, adenoviruses) and RNA viruses (enterovirus, influenza virus, respiratory syncytial virus, human metapneumovirus, rhinovirus, Toscana virus, West Nile virus, lymphocytic choriomeningitidis virus, dengue virus, chikungunya virus).

## Materials and Methods

### 1. External controls (ECs)

Important criteria for the design of ECs include (i) the possibility to use quantified or semi quantified controls (in order to manage the detection level of the diagnostic test used), (ii) the possibility to distinguish amplicons obtained from ECs from those obtained from the detected pathogen (in order to detect false positives due to accidental amplification of EC) and (iii) the use of relevant nucleic acids (*ie*, RNA and DNA molecules for RNA and DNA viruses, respectively).

**Figure 1:**
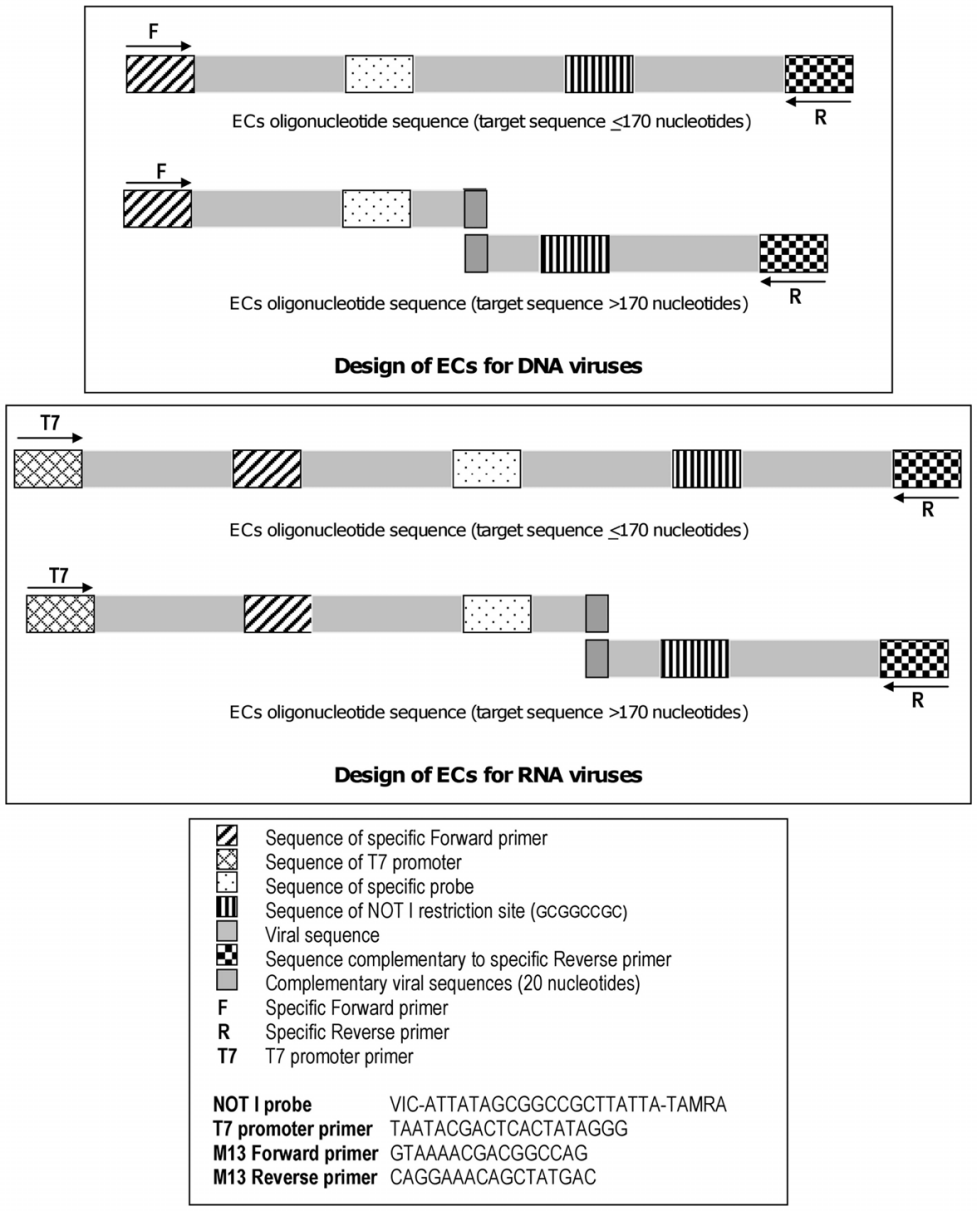
Design of synthetic external controls and sequences of NOT I probe,T7 promoter primer and M13 primers.

Simple protocols for preparing plasmid DNA or synthetic RNA quantified controls are described in Supporting Information S1 and schematised in figure 1. Such controls include specific sequences for the hybridisation of detection primers and probe, but also an exogenic “Not I” sequence (detectable by a specific probe or cleavable by the Not I restriction enzyme).

### 2. Internal controls (ICs)

#### 2a. rt-PCR assays for the detection of T4 and MS2 bacteriophages

The criteria to be addresses regarding ICs were: (i) to monitor all steps of the diagnostic procedure (extraction, RT, PCR); (ii) to be amenable for DNA and RNA viruses ; (iii) to rely on a detection system specific of the phage(s) to avoid complex molecular constructs; (iv) to be usable in either simplex or multiplex format, and in one-step or two-step RT-PCR reactions.

We used freeze-dried *E. coli* Enterobacteria phage T4 (T4) and Enterobacteria phage MS2 (MS2) obtained from the American Type Culture Collection (ATCC ref. 11303-B4 & 15597-B1, respectively). Protocols for real time PCR detection of phages TA and MS2 were elaborated in various formats and are described in Supporting Information S2. Briefly, primers and probes targeting T4 phage (T4F CCATCCATAGAGAAAATATCAGAACGA, T4R TAAATAATTCCTCTTTTCCCAGCG, T4probe *VIC*-AACCAGTAATTTCATCTGCTTCTGATGTGAGGC-*TAMRA)* and MS2 phage (MS2F CTCTGAGAGCGGCTCTATTGGT, MS2R GTTCCCTACAACGAGCCTAAATTC, MS2probe *VIC*-TCAGACACGCGGTCCGCTATAACGA-*TAMRA*) were designed from genomic sequences. Optimised oligonucleotide concentrations were 10pmol for primers and 4pmol for probes for both T4 and MS2 detection assays.

#### 2b. Spiking and validation procedures

A suspension of T4 and MS2 phages was used for spiking clinical samples. Both phages were diluted to provide a similar level of detection by rt-PCR.

The following format was used:

- Ten microlitres of a solution “T4 + MS2” were added to 200µL of sample
- The dilution of phages was adjusted to provide a Cycle threshold (Ct) at 30 for both the T4 and MS2 real time PCR assays upon extraction and amplification of 200µL of PBS spiked with the phage mix.

The influence of spiking on the performance of viral nucleic acid extraction was evaluated by comparing detection of enterovirus (echovirus 30) and cytomegalovirus (CMV) in serial dilutions of cell cultures supernatant spiked or not spiked with phages (see Supporting Information S3).

Similarly, the influence of spiking on the sensitivity of rt-PCR detection was evaluated by comparing detection of enterovirus (EV) and CMV in serial dilutions of cell culture supernatant media and a series of positive clinical samples spiked with T4-MS2 mix in either Two-step or One-step rt-PCR format (see Supporting Information S4).

### 2. Routine application

#### 2a. Clinical samples

From January 2007 to May 2008, a total of 8,950 clinical specimens received in the virology laboratory of the Public Hospitals of Marseille with a demand of PCR detection for at least one of the viruses listed in Table 1 were included in the study.

**Table 1:** PCR systems used for the detection of DNA or RNA viruses.

Virus	Authors	Reference
**Herpes Simplex Virus (HSV)**	Kessler et al. [9]	J Clin Microbiol 2000
**Varicella Zona Virus (VZV)**	in house	Unpublished
**Cytomegalovirus (CMV)**	Griscelli et al. [10]	J Clin Microbiol 2001
**Human Herpes Virus 6 (HHV6)**	Locatelli et al. [11]	J Clin Microbiol 2000
**Human Herpes Virus 8 (HHV8)**	Lallemand et al. [12]	J Clin Microbiol 2000
**Adenovirus**	Adenovirus R-gene™ (Argene)	/
**JC virus**	Whiley et al. [13]	J Clin Microbiol 2001
**BK virus**	Whiley et al. [13]	J Clin Microbiol 2001
**Parvovirus B19**	Abeham et al. [14]	J Virol Methods 2001
**Enterovirus**	Watkins-Riedel T et al. [15]	Diagn Microbiol Infect Dis. 2002
**Chikungunya virus**	Pastorino et al. [16]	J Virol Methods 2005
**Influenza A and B virus**	Van Elden et al. [17]	J Clin Microbiol 2001
**Respiratory Syncitial Virus (RSV)**	Van Elden et al. [18]	J Clin Microbiol 2003
**Choriomeningitis Lymphocytic virus (CML)**	in house	Unpublished
**Rhinovirus**	Garbino et al. [19]	Am J Respir Crit Care Med 2004
**Toscana virus**	Pérez-Ruiz et al. [20]	J Clin Virol 2007
**West Nile Virus (WNV)**	Lanciotti et al. [21]	J Clin Microbiol 2000
**Dengue virus**	Leparc-Goffart et al. [22]	J Clin Virol 2009

The approval of the relevant Ethics Committee (IFR48, Marseilles, France) was obtained for investigating the benefit of using phage spiking, but individual consent from patients was not required since French national regulations under the term of Biomedical Research (Loi Huriet-Sérusclat (loi 881138)) indicate that the signature at the hospital entrance office warrants that all samples taken during hospitalization for diagnostic purposes are accessible for research (excluding human genetic research) without the specific consent of the patient.

#### 2b. Spiking and rt-PCR assays

A 200µl volume of each 8,950 clinical specimens was spiked (according to the procedure described above) before extraction which was performed onto the MagNA Pure LC instrument (Roche) using the High Pure Viral Nucleic Acid Kit (DNA extraction) or the MagNA Pure LC RNA isolation High Performance kit (RNA extraction) according to manufacturer's recommendations. CSF, amniotic fluids, biopsy tissues, effusions and aqueous humor were processed for DNA+RNA extraction using the BioRobot EZ1 with the Virus Mini Kit v2.0 (both from Qiagen). Reverse transcription was performed for RNA viruses using the TaqMan Reverse Transcription Reagents kit (Roche) and random hexanucleotides as per manufacturer's instructions. For each specimen: *(i)* rt-PCR reactions were carried out according to medical prescription (Table 1), and *(ii)* distinct rt-PCR reactions for detection of T4 or MS2 were performed under a 15 µL reaction format (7,5 µL of mastermix, 3 pmol of each primer and 1,2 pmol of probe) and a standard cycling protocol (50°C for 2 min, 95°C for 10 min and 45 cycles 95°C for 15 sec, 60°C for 1 min).

#### 2c. Interpretation of results

For each series of T4 and MS2 rt–PCR, the mean Ct value and the standard deviation within the series were calculated. Each individual reaction was subsequently analysed as follows:

- If the Ct value was equal to or lower than the mean Ct value of the series +1SD, it was recorded as “correct detection of the phage” (CDP), and associated with the absence of detectable inhibitor or technical problem while processing the corresponding sample.
- If the Ct value was higher than the mean Ct value of the series +1SD (or undetectable), it was recorded as “inefficient detection of the phage” (IDP), and associated with the presence of amplification inhibitor(s) or technical problem while processing the corresponding sample.
  - When IDP was associated with a positive PCR (detection of a pathogen), this result was validated despite the presence of inhibitors (this would not apply to the case in which quantification of viral load is necessary).
  - When IDP was associated with negative PCR detection results, a new assay was performed using a tenfold dilution of the nucleic acid extract. All negative results were considered unresolved (UNR). Positive results were validated.

## Results

### 1. Optimization and validation of procedures

The detection of EV and CMV in serial dilutions of supernatant cell culture media or in series of positive clinical samples spiked with T4-MS2 mix was performed and the comparison of the Ct values showed that neither the addition of the phage mix itself (Wilcoxon test, p = 0.18 for CMV and 0.45 for EV), nor the presence of phage-specific primers and probe in the reaction mix (Wilcoxon test, p = 0.77 for CMV and 0.18 for EV) did interfere with the Ct value associated with viral detection in Two-step (Table 2 and 3) and One-step rt-PCR.

**Table 2:** Influence of spiking on the performance of viral nucleic acid purification.

	Virus	Unspiked specimen (A)	IC spiked specimen (B)	Wilcoxon test
**a**	**CMV 10^−3^**	33.6	33.8	NS
	**CMV 10^−4^**	36.3	36.6	NS
	**CMV 10^−5^**	39.6	39.4	NS
**b**	**EV 10^−3^**	30.1	30.6	NS
	**EV 10^−4^**	33.7	33.8	NS
	**EV 10^−5^**	38.2	37.8	NS

Cycles Thresholds (Ct) of serial dilutions of supernatant media of CMV (a) or EV (b) cell cultures unspiked (A) or spiked (B) with T4-MS2 IC were presented here. Each samples were tested in triplicate (the mean value was calculated). The Wilcoxon test were used to compared Ct values (NS = non significative).

**Table 3:** Influence of spiking on the performance of viral nucleic acid detection.

	Virus	CMV or EV detection system alone (A)	Multiplex system (B)	Wilcoxon test
**a**	**CMV 10^−3^**	33.9	33.8	NS
	**CMV 10^−4^**	36.7	37.0	NS
	**CMV 10^−5^**	39.8	39.5	NS
**b**	**EV 10^−3^**	30.7	30.8	NS
	**EV 10^−4^**	33.9	33.8	NS
	**EV 10^−5^**	38.3	37.9	NS

Serial dilutions of supernatant media of CMV (a) or EV (b) cell cultures spiked with T4-MS2 IC were tested either with the CMV or EV detection system alone (A) or the multiplex system (B). All cycles Thresholds (Ct) were presented here. Each samples were tested in triplicate (the mean value was calculated). The Wilcoxon test were used to compared Ct values (NS = non significative).

### 2. Phage spiking in routine rt-PCR virological diagnosis

**Figure 2:**
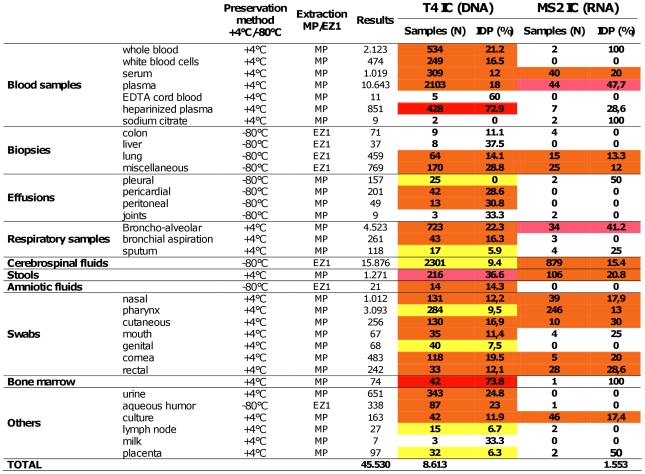
Results of the routine hospital use of bacteriophage ICs. MP (MagNA Pure LC instrument extraction), EZ1 (BioRobot EZ1 extraction). N (Number of samples), IDP (Inefficient detection of the phage). Results represent the number of PCR tests performed for each type of samples. Series with less than 10 samples are no highlighted. Samples with less than 10% of IDPs are highlighted in yellow, between 10 and 30% of IDPs in orange, between 30 and 50% of IDPs in pink and more than 50% of IDPs in red.

During the period of study, 8,950 clinical samples were spiked with phages and tested: 7,397 for the presence of DNA viruses only, 337 for the presence of RNA viruses only and 1,216 for both, corresponding to a total of 45,530 results transmitted to the prescribers (see details in Figure 2).

Inhibitors could be detected for all types of samples, but with highly variable rates (analysis performed for series including a minimum of 10 samples): less than 10% in the case of pleural effusions and sputum samples, genital and pharynx swabs, lymph nodes and placentas; 10% to 30% for EDTA whole blood, white blood cells, sera, biopsies, pericardial and peritoneal effusions, bronchial aspiration, CSF, amniotic fluids, others swabs, urine, aqueous humor and culture cells; 30% to 50% for plasma, BAL and stools; more than 50% for heparinized plasma and bone marrow.

IDPs were significantly more frequently detected for MS2 (RNA) than T4 (DNA) in plasma, BAL and CSF samples (p<0.05, Khi2 test, Yates corrected). Conversely, IDPs were significantly more frequently detected for T4 than MS2 in stools (p<0.05, Khi2 test, Yates corrected).

Finally, it was noted that clinical samples stored at −80°C prior extraction exhibited an IDP rate lower than 30%.

## Discussion

Rt-PCR and rt-RT-PCR techniques are now widely used for the molecular diagnosis of viral infections. Our study was focused on the importance of quality controls for such diagnostic assays. We proposed a simple protocol for synthesizing specific external controls which combines standard techniques for obtaining quantified DNA or RNA positive controls. Importantly, these controls were designed to include an extrinsic sequence that contains a (very rare) cutting site for the NotI restriction enzyme. This provides a simple tool for using adapted and reproducible amounts of positive controls, and also for identifying PCR contamination due to carry over of the positive control. This detection can be performed by real time amplification using the specific Not I probe (Figure 1) or following gel analysis after restriction by NotI.

Dreier *et al.* (2005,2006), Rolfe *et al.* (2007) and Gerriets *et al.* (2008) previously evaluated the use of MS2 or T4 ICs for testing limited series of sera, cerebrospinal fluids and stools [4], [5], [6], [7]. Our study confirms that the performance of ‘home made’ tests can be significantly improved by the used of phage-based internal controls, but, most importantly, shows that such controls can be used for routine virological diagnosis and usable for a variety of clinical samples. Here, clinical samples were spiked with both T4 and MS2 phages, allowing the detection of inhibitors for both DNA and RNA viruses. Thirty-six different types of clinical samples were tested (including various blood samples, cerebrospinal fluids, stools, respiratory samples, swabs, biopsies or effusion fluids) and a large number of samples were tested in the context of an hospital routine molecular virology laboratory. The use of phages as internal controls proved to be extremely versatile and could be adapted to a broad range of methods and pathogens. It was validated for both PCR and RT-PCR real time techniques, in simplex or multiplex format and, in the case of RT-PCR assays, one-step or two-step amplification formats. In addition, whilst the current report relies on probe-based real time amplification techniques, the method could also be conveniently adapted to a real time SYBR Green detection assay [8]. This is important and suggests that a strategy including phage-based internal controls can be implemented in diagnostic laboratories irrespective of the technical characteristics of the amplification methods used for routine tests. In our experience, the simplex format strategy (*i.e.* based on testing phages and viral pathogens in distinct amplification reactions) proved to be the most simple and cost-effective for routine molecular diagnosis since it does not require the specific development of multiplex reactions and relies on a unique control reaction for DNA viruses and another for RNA viruses.

Our study identified different frequencies of inhibitors according to the nature of the clinical samples. In samples such as CSF, sera or pharynx swabs, inhibitors were identified in ∼10% and ∼15–20% for DNA and RNA virus detection, respectively. These values are unexpectedly high, and imply that a significant number of samples with results believed to be “negative” in the absence of an internal control should be considered “unresolved”. Specific samples such as heparinized blood (72,9% of inhibitors for DNA virus detection), bone marrow (73.8% of inhibitors for DNA virus detection) and stools (36.6% and 20.8% of inhibitors for DNA and RNA viruses detection, respectively) may also benefit from the detection of inhibitors and the identification of “unresolved” tests.

It should be noted that the frequency of amplification inhibition not only relates to the nature of the sample tested, but is also intrinsically linked with the technical protocols used for the extraction and amplification of nucleic acids. In our experience, no major differences were observed when different silica column- or magnetic beads- based extraction techniques, or different commercialized PCR or RT-PCR kits, were tested (data not shown). However, a detailed analysis may reveal that specific techniques give better results when used for testing specific samples. We suggest that, in the future, phage-based internal controls may constitute cost-effective tools with which to measure the frequency of amplification inhibition in specific samples. Estimation of this parameter may become a major criterion for the evaluation of extraction (and to a lesser extent amplification) techniques.

Finally, in the context of diagnostic virology laboratories, our study shows that standard extraction and amplification techniques used for the molecular diagnosis of human pathogens led to a significant proportion of ‘unresolved’ results, which cannot be identified if an internal control is not used. In the absence of an internal control, such samples are commonly identified as ‘negative’, which is with hindsight incorrect: in our hands, detecting amplification inhibition using phages as internal controls, and testing tenfold dilutions of the nucleic acid extracts demonstrated that some of these samples were actually positives. This cost-effective and convenient strategy can therefore be used for enhancing the quality of routine molecular diagnosis, but it may also be adapted in other contexts such as testing of large numbers of animal or environmental samples.

## Supporting Information

Supporting Information S1 Preparation of DNA and RNA synthetic ECs.
(DOC)

Supporting Information S2 Real time PCR assays and optimisation of bacteriophage detection system.
(DOC)

Supporting Information S3 Influence of spiking on the performance of viral nucleic acid extraction.
(DOC)

Supporting Information S4 Detection of phages and pathogens in a one-reaction multiplex format.
(DOC)
