# Supplementary material for: RNA and DNA Bacteriophages as Molecular Diagnosis Controls in Clinical Virology: A Comprehensive Study of More than 45,000 Routine PCR Tests
Source: PLoS One. 2011 Feb 9;6(2):e16142. doi: 10.1371/journal.pone.0016142 (PMC3036576; doi:10.1371/journal.pone.0016142)
Supplement: Supporting Information S1 — Preparation of DNA and RNA synthetic ECs. (DOC) [file pone.0016142.s001.doc]

**Supporting Information S1: preparation of DNA and RNA synthetic ECs**

- *DNA synthetic ECs*

In the case of DNA targeted sequences shorter than 170 nucleotides, a long, unique, primer was synthesized (Figure 1). An amplification reaction was prepared, that included (final volume: 50µL): the long synthesized primer (5µL at 10µM), the standard forward and reverse primers used for diagnosis (1µL at 10µM of each primers), 1µL of dNTP (10mM), 5µL of buffer (10X), 0.2µL of *Taq* polymerase (Invitrogen, Cergy Pontoise, France). The cycling program was 94°C for 5min, 35 cycles at 94°C for 30sec, 45°C for 30sec, 72°C for 30sec and 72°C for 10min.

In the case of DNA targeted sequences of 170 nucleotides or more, two long primers (of forward and reverse polarity, respectively) including a 20 nucleotide complementary sequence at their 3' end were synthesized (Figure 1). An amplification reaction was prepared, that included (final volume: 50µL): the long forward and reverse primers (5µL at 10µM of each), the standard forward and reverse primers used for diagnosis (1µL at 10µM of each primers), 1µL of dNTP (10mM), 5µL of buffer (10X), 0.2µL of *Taq* polymerase (Invitrogen, Cergy Pontoise, France). The cycling programme was the same as detailed above.

In all cases, the amplification product was purified using the PCR purification kit (Millipore, Molsheim, France) and cloned using the TA cloning kit (Invitrogen, Cergy Pontoise, France). Individual clones were selected and sequenced using the M13 primers (Figure 1) for confirmation of the cloned sequence prior to subsequent medium scale production and purification (Qiaprep spin miniprep, Qiagen S.A, Courtaboeuf, France). The DNA concentration was determined by spectrometry (NanoDrop 1000 Thermo Scientific, Wilmington, USA) and the final quantification (number of copies per mL) was calculated as follows:

N(copies/µl) = [Concentration (µg/ml DNA).10^-3^ x (6,022.10^23^molecule/mol)] / [(nucleotide number x (2x330g/mol) x 10^6^]

- *RNA synthetic ECs*

A similar strategy was used. In the case of RNA targeted sequences shorter than 170 nucleotides, a unique primer was synthesized (Figure 1). An amplification reaction was prepared, that included (final volume: 50µL): the synthesized primer (5µL at 10µM), a standard forward primer designed within the T7 promoter sequence (1µL at 10µM), the standard reverse primer used for diagnosis (1µL at 10µM), 1µL of dNTP (10mM), 5µL of buffer (10X), 0.2µL of *Taq* polymerase (Invitrogen, Cergy Pontoise, France). The cycling programme was identical to the one detailed above.

In the case of RNA targeted sequences of 170 nucleotides or more, two long primers (of forward and reverse polarity, respectively) including a 20 nucleotide complementary sequence at their 3' end were synthesized. The 5' terminus of the forward primer included the sequence of the T7 RNA polymerase promoter (Figure 1). An amplification reaction was prepared, that included (final volume: 50µL): the long forward and reverse primers (5µL at 10µM of each), a standard forward primer designed within the T7 promoter sequence (1µL at 10µM) (Figure 1), the standard reverse primer used for diagnosis (1µL at 10µM), 1µL of dNTP (10mM), 5µL of buffer (10X), 0.2µL of *Taq* polymerase (Invitrogen, Cergy Pontoise, France). The cycling programme was identical to the one detailed above.

PCR products were used for synthesis of RNA using the T7 Mega short script kit (Applied Biosystems/Ambion, Austin, USA): 2µL of ATP, CTP, GTP, UTP, 2µL of buffer, 8µL of PCR product, 2µL of Mix Enz and incubation for 5 hours at 37°C. Synthetic RNAs were further treated with DNase and purified using the Megaclear purification Kit as per manufacturer’s instructions. The RNA concentration was determined by spectrometry at 260nm (NanoDrop 1000 Thermo Scientific, Wilmington, USA) and the final quantification (number of copies per mL) was calculated as follows:

N(copies/µl) = [Concentration (µg/ml RNA).10^-3^ x (6,022.10^23^molecule/mol)] / [(nucleotide number x 340g/mol) x 10^6^]
