# Supplementary material for: RNA and DNA Bacteriophages as Molecular Diagnosis Controls in Clinical Virology: A Comprehensive Study of More than 45,000 Routine PCR Tests
Source: PLoS One. 2011 Feb 9;6(2):e16142. doi: 10.1371/journal.pone.0016142 (PMC3036576; doi:10.1371/journal.pone.0016142)
Supplement: Supporting Information S2 — Real time PCR assays and optimisation of bacteriophage detection system. (DOC) [file pone.0016142.s002.doc]

**Supporting Information S2: Real time PCR assays and optimisation of bacteriophage detection system**

Tenfold serial dilutions in Hanks medium of T4 or MS2 bacteriophages were used for subsequent analyses. Extraction of T4 DNA and MS2 RNA was performed using the MagNA Pure LC instrument (Roche Diagnostic Systems, Inc., Branchburg, N.J) and the High Pure Viral Nucleic Acid Kit (DNA extraction) or the MagNA Pure LC RNA isolation High Performance kit (RNA extraction). Reverse transcription of MS2 RNA was performed using the TaqMan Reverse Transcription Reagents kit (Roche) and random hexanucleotides according to the manufacturer's recommendations. Real time PCR reactions were performed using a Stratagene Multiplex Quantitative PCR System Mx3005 P thermocycler (Agilent), the qPCR MasterMix-No Rox kit (Eurogentec) and a standard cycling protocol recommended by the manufacturer (50°C for 2 min, 95°C for 10 min and 45 cycles 95°C for 15 sec, 60°C for 1 min). Real time PCR assays has been optimised using a strategy based on systematic experiments with various concentrations of primers (5, 10, 15, 20, 25 pmol) and probe (2, 4, 6, 8, 10 pmol)
