# Supplementary material for: RNA and DNA Bacteriophages as Molecular Diagnosis Controls in Clinical Virology: A Comprehensive Study of More than 45,000 Routine PCR Tests
Source: PLoS One. 2011 Feb 9;6(2):e16142. doi: 10.1371/journal.pone.0016142 (PMC3036576; doi:10.1371/journal.pone.0016142)
Supplement: Supporting Information S3 — Influence of spiking on the performance of viral nucleic acid extraction. (DOC) [file pone.0016142.s003.doc]

**Supporting Information S3: influence of spiking on the performance of viral nucleic acid extraction**

This was tested as follows: 200 µL of serial dilutions of supernatant medium from MRC5 or Vero cell cultures infected with CMV and echovirus 30, respectively, were spiked using either 10µL of the T4-MS2 mix or 10µL of PBS; DNA (from CMV culture) and RNA (from echovirus 30 culture) extractions and RNA reverse transcription were performed as described above. Samples were subsequently tested in triplicate for the presence of CMV or echovirus 30. References of primers and probes used for detection of CMV and echovirus 30 are provided in Table 1.
