# Supplementary material for: RNA and DNA Bacteriophages as Molecular Diagnosis Controls in Clinical Virology: A Comprehensive Study of More than 45,000 Routine PCR Tests
Source: PLoS One. 2011 Feb 9;6(2):e16142. doi: 10.1371/journal.pone.0016142 (PMC3036576; doi:10.1371/journal.pone.0016142)
Supplement: Supporting Information S4 — Detection of phages and pathogens in a one-reaction multiplex format. (DOC) [file pone.0016142.s004.doc]

**Supporting Information S4: Detection of phages and pathogens in a one-reaction multiplex format**

- *Two-Step rt-PCR*

***Cell cultures:*** 200 µL of serial dilutions (10^-3^ to 10^-5^) of supernatant media from MRC5 and Vero cell cultures infected by human cytomegalovirus (CMV) and echovirus 30, respectively, were spiked as described above. DNA (from CMV culture), RNA (from echovirus 30 culture) extractions and RNA reverse transcription were performed as described above. For each spiked sample, two series of PCR reactions were tested in triplicate: one for the detection of CMV or echovirus 30 (2 primers and 1 probe), and the other in a multiplex format for the detection of both CMV and T4 on the one hand or both echovirus 30 and MS2 on the other (4 primers and 2 probes). The optimized protocol used 25µL of mastermix, 10 pmol of each primer and 4 pmol of probe in a 50µL final volume and a standard cycling protocol (see above).

***Clinical samples:*** 200 µL of clinical samples submitted for enterovirus (538 specimens: cerebrospinal fluid (CSF), 452; pharyngeal sample, 59; stool, 13; heparinized blood, 6; diverse, 8) and CMV (206 specimens: broncho-alveolar liquid (BAL), 5; CSF, 58; heparinized blood, 58; urine, 10) real-time PCR detection were spiked as described above. DNA and RNA extractions and RNA reverse transcription were performed as described above. Samples were tested in triplicate as described above for cell cultures. The number of positive detections was subsequently compared between both series.

- *One-Step rt-PCR*

In addition to these protocols a one-step RT-PCR multiplex procedure was tested for the West Nile virus (WNV), *i.e.* reverse transcription and PCR were performed in a single tube for the simultaneous detection of MS2 phage and West Nile virus. 200 µL of supernatant medium from Vero cell cultures infected with WNV were spiked using 10µL of the phage mix. RNA extraction was performed as described above. RT-PCR was performed using the SuperScript one-step RT-PCR Platinum® *Taq* mastermix (Invitrogen, Cergy Pontoise, France), MS2 primers and probe, WNV specific primers and probe (Table 1 for references) and a standard cycling protocol following the manufacturer's recommendations (48°C for 30 min, 95°C for 10 min, 50 cycles at 95°C for 15 sec, 60°C for 1 min).
